# Cost effectiveness of a pentavalent rotavirus vaccine in Oman

**DOI:** 10.1186/1471-2334-14-334

**Published:** 2014-06-17

**Authors:** Salah Thabit Al Awaidy, Berhanu G Gebremeskel, Idris Al Obeidani, Said Al Baqlani, Wisam Haddadin, Megan A O’Brien

**Affiliations:** 1 Office of HE of Health Affairs, Ministry of Health, Muscat, Oman; 2 Rutgers School of Public Health, The State University of New Jersey, Piscataway, NJ, USA; 3 Department of Communicable Disease Surveillance & Control, DGHA, Ministry of Health, Muscat, Oman; 4 Central Public Health Laboratory, DGHA, Ministry of Health, Muscat, Oman; 5 MSD EEMEA, Dubai, United Arab of Umirates; 6 Global Health Outcomes, Merck & Co. Inc, West Point, PA, USA; 7 Communicable Disease Advisor to Health Affairs, Office of Undersecretary of Health Affairs, Ministry of Health, Post Box 393, Muscat Postal Code 113, Sultanate of Oman, Oman

**Keywords:** Rotavirus, Vaccine, Cost effectiveness, Oman, Markov model

## Abstract

**Background:**

Rotavirus gastroenteritis (RGE) is the leading cause of diarrhea in young children in Oman, incurring substantial healthcare and economic burden. We propose to formally assess the potential cost effectiveness of implementing universal vaccination with a pentavalent rotavirus vaccine (RV5) on reducing the health care burden and costs associated with rotavirus gastroenteritis (RGE) in Oman

**Methods:**

A Markov model was used to compare two birth cohorts, including children who were administered the RV5 vaccination versus those who were not, in a hypothetical group of 65,500 children followed for their first 5 years of life in Oman. The efficacy of the vaccine in reducing RGE-related hospitalizations, emergency department (ED) and office visits, and days of parental work loss for children receiving the vaccine was based on the results of the Rotavirus Efficacy and Safety Trial (REST). The outcome of interest was cost per quality-adjusted life year (QALY) gained from health care system and societal perspectives.

**Results:**

A universal RV5 vaccination program is projected to reduce, hospitalizations, ED visits, outpatient visits and parental work days lost due to rotavirus infections by 89%, 80%, 67% and 74%, respectively. In the absence of RV5 vaccination, RGE-related societal costs are projected to be 2,023,038 Omani Rial (OMR) (5,259,899 United States dollars [USD]), including 1,338,977 OMR (3,481,340 USD) in direct medical costs. However, with the introduction of RV5, direct medical costs are projected to be 216,646 OMR (563,280 USD). Costs per QALY saved would be 1,140 OMR (2,964 USD) from the health care payer perspective. An RV5 vaccination program would be considered cost saving, from the societal perspective.

**Conclusions:**

Universal RV5 vaccination in Oman is likely to significantly reduce the health care burden and costs associated with rotavirus gastroenteritis and may be cost-effective from the payer perspective and cost saving from the societal perspective.

## Background

Rotavirus is the leading cause of severe gastroenteritis in children under age 5. The virus is responsible for more than 453,000 (range: 420,000-494,000) deaths each year, of which more than 90% occur in low-resource countries [1-3]. Virtually all children will have acquired a rotavirus infection by the age of 5, regardless of socioeconomic status [4,5]. Although occasionally asymptomatic, a rotavirus infection often results in diarrhea, vomiting, fever and/or lethargy lasting from a few days to a few weeks [6,7]. Severe episodes of rotavirus gastroenteritis (RGE) are more likely to involve a combination of these symptoms, leading to dehydration if left untreated. Although deaths are rare in industrialized countries, the health care burden of RGE-related hospitalizations and emergency department (ED) visits is substantial [8-10]. The impact of the rotavirus on families of infected children can be considerable as well [11].

Vaccines to prevent RGE were developed subsequent to the recognition that wild-type rotavirus infection induces immunity against subsequent disease [5,12-14]. Primary rotavirus infections provide substantial protection against gastroenteritis caused by the same serotype and against severe disease, regardless of serotype [15]. Since 2006, the World Health Organization (WHO) has recommended two oral rotavirus vaccines (RotaTeq® [RV5], Merck & Co.; and Rotarix™; GlaxoSmithKline Vaccines). Both have positively demonstrated safety and efficacy in clinical trials and effectiveness profiles, as well as impact in real world settings [15,16].

In the Sultanate of Oman, data on the burden of rotavirus were previously analyzed in 2009 by Al Awaidy et al. [17] In an effort to assess the need for a rotavirus vaccine, the Oman Ministry of Health had implemented a nationwide surveillance of severe childhood diarrhea in under five children at sentinel hospitals in 2006, using the procedures outlined in the WHO generic protocol for rotavirus surveillance [17]. Using this surveillance-based data, Al Awaidy et al. conducted a comprehensive burden assessment in Oman and concluded that rotavirus was the single most common cause of diarrhea in young children, accounting for one-half of all acute gastroenteritis hospitalizations. Furthermore, an estimated 3,300 rotavirus-associated hospitalizations occur annually in Oman. An additional 11,500 children receive outpatient treatment for rotavirus at total annual costs exceeding 2.6 million United States dollars (USD) [17]. The health care and economic burden associated with rotavirus in Oman suggested that the introduction of a safe and effective vaccine can provide considerable benefits from both payer and societal perspectives.

The purpose of the current study was to assess the potential cost-effectiveness of implementing universal RV5 vaccination in Oman, and provide policy makers with evidence-based information to support the introduction of rotavirus vaccination in the routine infant immunization program.

## Methods

The cost-effectiveness analysis was based on a Markov model developed in Microsoft Excel [18]. The model evaluated the cost-effectiveness of universal RV5 vaccinations against RGE by tracking a hypothetical birth cohort of 65,500 children divided into two cohorts: with and without vaccination during the first 5 years of life. Although children can acquire multiple rotavirus infections, the first two infections are believed to be the most severe based on prospective observational epidemiological studies [5]. Therefore, the model allowed children to contract up to two symptomatic episodes of RGE, after which all subsequent infections were assumed to be asymptomatic.

**Figure 1:**
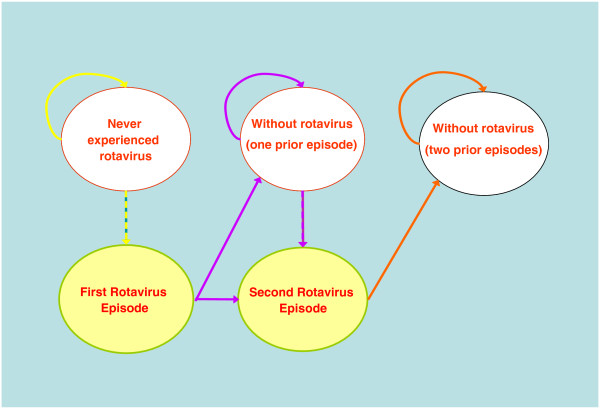
**Description of the RotaTeq™ Markov model.** The model demonstrating the transition cycle for children to move over a Markov cycle. ***Note:*** Markov cycles are monthly through 6 months, quarterly through year 5.

The model allowed for children to move between different health states over a discrete period of time, known as a Markov cycle (Figure 1) [19]. For the first 6 months of life, the Markov model used monthly cycles. Quarterly cycles were employed after 6 months and through 5 years. Transition probabilities reflected the likelihood of moving from one health state to another. Health status reflected whether the child had ever experienced a rotavirus infection, the number of prior rotavirus infections, serotypes causing the episodes and whether the child survived the illness.

This manuscript is a cost effectiveness analysis based on a Markov model and did not involve any experimental research at all, and does not require approval by IRB. The epidemiological inputs from Al Awaidy et al 2009 (reference 17), which is an observational prospective surveillance study, had all the necessary ethical approvals.

### Analytic perspective and outcome measures

The cost effectiveness analysis assessed the impact of RV5 vaccinations administered orally in three doses within the first year of life at ages 2, 4, and 6 months on the health care and cost burden associated with RGE among a hypothetical birth cohort of Omani children age 5 and under. The impact of adding the RV5 vaccine to routine infant immunizations was quantified as the net cost per vaccine, as well as the cost per quality-adjusted life year (QALY) gained from the health care system and societal perspectives. The health care system perspective accounted only for direct medical care and vaccination program costs. The societal perspective included direct and indirect (e.g., parental work loss to care for children infected with the rotavirus), medical and non-medical (e.g., travel to/from health care facilities) costs.

The outcome measure of interest was the QALY gained by universal vaccination compared to no vaccination. The QALY is a standardized measure that is scaled from 0 to 1, with 0 signifying death and 1 signifying perfect health. The QALY incorporates morbidity and mortality and may be used to compare interventions across different diseases. The utility weights used to estimate the QALY for each episode of rotavirus were adapted from a study conducted in Canada and was based on standard measures that are used to assess utilities associated with different health states Brisson et al [20]. The benchmarks used to determine the cost effectiveness of universal vaccinations were based on recommendations by the WHO Commission on Macroeconomics and Health, classified as highly cost-effective if the cost per QALY gained is lower than the per capita gross domestic product (GDP), possibly cost-effective if the expenditure is less than 2-3 times the per capita GDP, and not cost-effective if the cost is higher than 3 times the per capita GDP [21,22]. In Oman, the per capita GDP in 2010 was 7,938 Omani rial (OMR) (20,640 USD; 1 OMR = 2.60 USD) [23].

### Model inputs

#### General overview

Table 1 shows the key model inputs with values for the base case scenario, including ranges for the one-way sensitivity analysis. All medical and non-medical care costs are expressed in OMR (and USD) and adjusted for inflation to 2010 OMR (and USD). The base case scenario assumed that 94% of children received three RV5 doses, 3% received two doses, and the remaining 3% received one dose, based on the immunization coverage levels of diphtheria, tetanus, and pertussis (DTaP), which are administered on the same schedule [24].

**Table 1:** Analysis model inputs

**Parameter**	**Base case value (Range for one-way sensitivity analysis)**	**Source**
Birth cohort	65500	Birth Registry DG-PL
Deaths	6 (4.8,7.2)	Al Awaidy 2009, Parashar 2004
Hospitalizations	4279 (3423, 5135)	Al Awaidy 2009
ED Visits	3294 (2,635, 3,953)	Al Awaidy 2009, Howidi 2012
Office visits	14,838 (11870, 17,806)	Al Awaidy 2009, Parashar 2003 &2006
Average hospital length of stay	3 days	Al Awaidy 2009
Distribution of health care contacts by type		Al Awaidy 20091
**Vaccine efficacy (assumed rate reduction) for complete series**		
Deaths and hospitalizations	88%	Vesikari et al. 2006
Office visits	86%	Vesikari et al. 2006
Parental work loss		Howidi, 2012
Days of work loss for episodes requiring death/hospitalization	2	Assumption
Days of work loss for episodes requiring ED visits	1	Assumption
Days of works loss for episodes requiring office visits	0.50	Assumption
Days of works loss for episodes requiring no care	0.50	Assumption
Percentage of working parents who missed work for episodes involving deaths/hospitalizations	100%	Assumption
Percentage of working parents who missed work for episodes involving outpatient visits and no care	100%	Assumption
Vaccine Coverage	94%: 3 doses, 3%: 2 doses, 3%: 1 dose	WHO
**Health care and vaccination costs,OMR [USD]**		
Hospital day	71 (57, 85) [184.6 (148.2, 221)]	Al Awaidy 2009
ED visit	27 (21.6, 32.4) [70.2 (56.16,84.24)]	Al Awaidy, 2009
Office visit	27 (21.6, 32.4) [70.2 (56.16,84.24)]	Al Awaidy 2009
Day of missed work	11.40 [29.64]	
Direct non-medical care for hospitalizations or deaths	12.5 [32.5]	Assumption
Direct non-medical care for ED visits	7.5 [19.5]	Assumption
Direct non-medical outpatient visits and no care	7.5[19.5]	Assumption
Price per dose	7.69 [19.994]	
Administration fee per dose	0	Assumption
QALY decrement per rotavirus episode	0.0058 (0.0022, 0.0133)	Brisson et al. 2010

ED = emergency department; OMR = Omani rial; USD = United States dollars; QALY = quality-adjusted life year; DG-PL = Omani Birth Registry Director General of Planning; WHO = World Health Organization.

Exchange rate: 1 OMR = 2.60 USD.

The size of the birth cohort, based on the Omani Birth Registry Director General of Planning (DG-PL), was 65,500 [17]. The proportions of all rotavirus infections and the number of infections for each child were based on a unique prospective, field-based study conducted in Mexico by Velazquez et al. documenting all symptomatic and asymptomatic infections among 200 newborns [5]. In this landmark study, stool specimens were collected every 2 weeks for the first 2 years of the children's life, and these data provided a comprehensive description of the natural history of rotavirus infection in young children. In the Omani economic model, secondary infection rates were calculated assuming that nearly 80% of children under age 5 with a primary infection were at risk of contracting a second infection. The distribution of primary and secondary infections by age was based on the surveillance data from Oman [17].

The efficacy of the vaccine in reducing the number of RGE-related hospitalizations, ED and office visits and days of parental work loss for children receiving the vaccine was based on the results of the Rotavirus Efficacy and Safety Trial (REST) [15,25]. REST was a Phase III study conducted in 11 countries, enrolling approximately 70,000 healthy infants. Given that most children followed the protocol, there are very little data regarding the efficacy for those receiving only one or two doses. In addition, the majority of serotypes in the trial were G1-G4 and G9. Thus the efficacy for those with one and two vaccine doses was based on subsequent routine effectiveness studies and efficacy for additional serotypes was assumed to be slightly lower than the efficacy of the 3 dose series based on observational studies of the effectiveness of RV5 [25]. These assumptions are described in detail in Itzler et al [18].

#### Base case scenario

The base case scenario relied on surveillance data provided by Al Awaidy et al. for the number of hospitalizations and outpatient office visits, average hospital length of stay and average medical expenditures for hospitalizations, and outpatient visits. No information on ED visits was available in this publication and therefore, information from the Parental Burden Survey conducted in Abu Dhabi, a neighboring country with a similar health care system, was used to estimate that 14.7% of all health care encounters in Oman are ED admissions [26]. Death due to rotavirus infection was calculated based on a case fatality rate of 9 per 100,000 children (Table 1) [27].

For an RGE episode resulting in hospitalization or death, 100% of working parents were assumed to have missed an average of 2 days of work. For episodes involving ED and office visits, 100% of working parents were assumed to have missed an average of 1 and 0.50 days of work, respectively.

Direct non-medical costs were assumed to be 12.50 OMR (32.5 USD) and 7.50 (19.5 USD) OMR for rotavirus episodes resulting in hospitalization/death and all other episodes, respectively. The cost of a work day missed was estimated at 11.40 OMR (29.64 USD), and the price per vaccine dose at 7.69 OMR (19.99 USD).

The QALY lost for each RGE episode used in this study was 0.0058, based on the Health Utilities Index 2 (HUI2) for children and the EuroQol 5D (EQ5D) questionnaire for parents from the Measuring the Impact of Rotavirus Acute Gastroenteritis Episodes (MIRAGE) study conducted in Canada by Brisson et al. [20].

#### Sensitivity analyses

Various sensitivity analyses were performed to evaluate the impact of using different assumptions for health care utilization, as well as the QALY loss associated with RGE episodes. One-way sensitivity analyses were performed for RGE hospitalization frequency and costs, ED and outpatient visits, death and QALY lost assumptions.

Additional one-way sensitivity analyses of health care utilization were also conducted. Estimates for the number of deaths, hospitalizations and ED and outpatient visits were evaluated using three scenarios from payer and societal perspectives: 10%, 25% and 50% fewer than the base case scenario.

## Results

### Base case scenario

The model predicted that, with the implementation of universal vaccination, an 89% reduction in RGE-related deaths would occur. The universal vaccination program would also prevent 3,813 hospitalizations, 2,644 ED visits, 9,886 outpatient visits and 22,200 parental work days lost due to rotavirus over five years (89%, 80%, 67%, 74% reduction, respectively) (Table 2).

**Table 2:** Public health and economic impact of a universal vaccination program vs. no vaccination program for base case scenario

	**No vaccination program**	**Universal vaccination program**	**Net difference**	**Percent reduction**
**Events**				
Deaths	6	0.65	-5	-89.1%
Hospitalizations	4,279	466.06	-3,813	-89.1%
ED visits	3,294	650.01	-2,644	-80.3%
Outpatient visits	14,838	4,951.67	-9,886	-66.6%
Work loss days	30,068	7,867.45	-22,200	-73.8%
**Costs, OMR (USD)**				
Medical care costs	1,338,977 (3,481,340)	216,646 (563,279.6)	-1,122,331 (-2,918,061)	-83.8%
Hospitalization costs	871,083 (2,264,816)	73,043 (189,911.8)	-798,041 (-2,074,907)	-91.6%
ED visit costs	85,001 (221,002.6)	16,659 (43,313.4)	-68,342 (-177,689)	-80.4%
Outpatient visit costs	382,893 (995,521.8)	126,944 (330,054.4)	-255,949 (-665,467)	-66.8%
Direct non-medical costs	347,527 (903,570.2)	137,216 (356,761.6)	-210,311 (-546,809)	-60.5%
Indirect (work days lost) costs	336,533 (874985.8)	89,758 (233,370.8)	-246,776 (-641,618)	-73.3%
Societal costs	2,023,038 (5259,899)	443,619 (1,153,409)	-1,579,419 (-4,106,489)	-78.1%
Vaccination costs	0	1,465,752 (3,810,955)		
Net direct medical care costs	1,338,977 (3,481,340)	1,682,398 (4,374,235)	343,421 (892,894.6)	25.7%
Net societal costs	2,023,038 (5,259,899)	1,909,371 (4,964,365)	-113,667 (-295,534)	-5.6%

ED = emergency department; OMR = Omani rial; USD = United States dollars.

Exchange rate: 1 OMR = 2.60 USD.

In the absence of a vaccination program, RGE-related societal costs of care are projected to be 2,023,038 OMR (5,259,899 USD), including 1,338,977 OMR (3,481,340 USD) in direct medical costs, of which 65% was due to hospitalization. However, with the introduction of a universal vaccination program, the model predicts RGE-related net societal costs in Oman to be 1,909,371 OMR (4,391,553 USD) and 216,646 OMR (563,280 USD) in direct medical costs.

The cost effectiveness results of universal vaccination compared to no vaccination in a single birth cohort in Oman, from birth through age 5, showed that costs per QALY saved would be 1,140 OMR (2,964 USD) from the payer perspective, and cost saving from the societal perspective. Specifically, the analysis showed that the cost would be 13 OMR (33.8 USD) per case avoided and 90 OMR (234 USD) per hospitalization avoided from the societal perspective (Table 3).

**Table 3:** Cost-effectiveness results for universal vaccination vs. no vaccination in a single birth cohort in Oman from birth through age 5 (OMR)

**Cost effectiveness outcome**	**Payer perspective**	**Societal perspective**
Cost per case avoided, OMR (USD)	13 (33.8)	Cost saving
Cost per hospitalization avoided, OMR (USD)	90 (234)	Cost saving
Cost per QALY saved, OMR (USD)	1,140 (2,964)	Cost saving

OMR = Omani rial; USD = United States dollars; QALY = quality adjusted life year.

Exchange rate: 1 OMR = 2.60 USD.

### Sensitivity analyses

**Figure 2:**
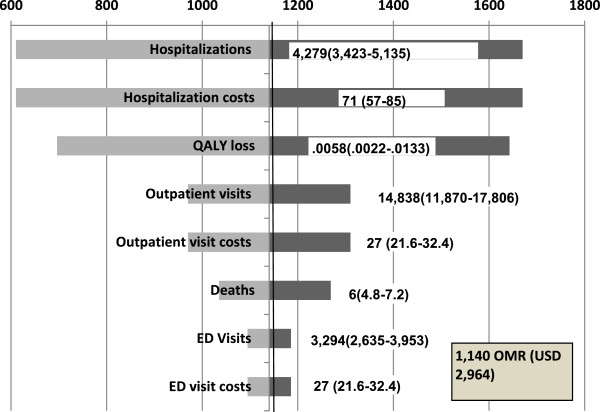
**Results of one-way sensitivity analysis: impact of variations in key parameters on ICER, payer perspective.** Values varied in sensitivity analysis indicated by the figures in the brackets. ICER = incremental cost effectiveness ratio; OMR = Omani rial; USD = United States dollars; QALY = quality-adjusted life year; ED = emergency department. Exchange rate: 1 OMR = 2.60 USD.

**Figure 3:**
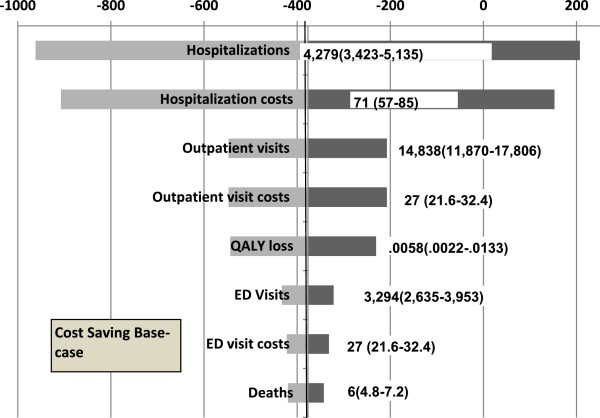
Results of one-way sensitivity analysis: impact of variations in key parameters on ICER, societal perspective.

The results of one-way sensitivity analyses from the payer and societal perspectives were most sensitive to the number of hospitalizations and hospitalization costs, followed by QALY loss per episode, number of outpatient visits and outpatient visit costs (Figures 2, 3). The model was least sensitive to the number of ED visits, ED visit costs.

Additional one-way sensitivity analyses of health care utilization parameters are reported in Table 4. When the vaccine administration fee is assumed to be 1.44 OMR/dose (3.74 USD), the incremental cost effectiveness ratios (ICERs) are 2,051 OMR (5,333 USD) and 534 OMR (1,388 USD), respectively, from payer and societal perspectives, which was higher, as expected, compared to the base case scenario (1,140 OMR [2,964 USD] per QALY saved). Sensitivity analyses assuming that health care utilization (for number of deaths, hospitalization, ED visits and outpatient visits) were lower by 10%, 25% and 50% of the base case scenario were cost effective from the payer and societal perspectives. ICERs had an increasing trend as expected, with higher costs per QALY saved using a 50% lower estimation in health care utilization than for the base case scenario. The sensitivity analysis assuming 50% fewer hospitalizations than the base case scenario resulted in ICERs of 2,465 OMR (6,409 USD) and 1,084 OMR (2,818 USD) from payer and societal perspectives, respectively, which were again higher than those from the payer perspective in the base case scenario, as expected. Finally, assuming death rates at 50% lower than the base case scenario resulted in ICERs of 1,527 OMR (1,970 USD), from the payer’s perspective.

**Table 4:** Additional one-way sensitivity analyses for health care utilization parameters

	**Payer perspective**	**Societal perspective**
**Scenario**	**Cost/QALY saved OMR (USD)**	**Cost/QALY saved OMR (USD)**
Base case scenario	1,140 (2,964)	CS
Vaccine administration Fee (1.44 OMR [USD 3.74])	2,051 (5,332.6)	534 (1,388.4)
Hospitalizations		
10% fewer than base case	1,405 (3,653)	CS
25% fewer than base case	1,802 (4,685.2)	353 (917.8)
50% fewer than base case	2,465 (6,409)	1,084 (2,818.4)
ED visits		
10% fewer than base case	1,163 (3,023.8)	CS
25% fewer than base case	1,197 (3,112.2)	CS
50% fewer than base case	1,254 (3,260.4)	CS
Outpatient visits		
10% fewer than base case	1,225 (3,185)	CS
25% fewer than base case	1,353 (3,517.8)	CS
50% fewer than base case	1,565 (4,069)	48 (124.8)
Deaths		
10% fewer than base case	1,201 (3,122.6)	CS
25% fewer than base case	1,306 (3,395.6)	CS
50% fewer than base case	1,527 (3,970.2)	CS

CS = cost saving; ED = emergency department; OMR = Omani rial; USD = United States dollars; QALY = quality adjusted life year.

Exchange rate: 1 OMR = 2.60 USD.

## Discussion

Based on a national surveillance study in the Sultanate Oman, Al Awaidy et al have demonstrated that RGE is the single most important cause of severe gastroenteritis in children accounting for almost one-half of all acute gastroenteritis hospitalizations, with 3,300 hospitalizations and more than 2.6million USD attributable to RGE. However, limited data exist on the potential impact of universal vaccination with rotavirus on the health care and economic burden in the Sultanate of Oman. The current study assessed the projected impact of universal vaccination with RV5 on the health care and economic burden associated with rotavirus infections in Oman. Results indicate that the introduction of a universal RV5 vaccination in Oman has the potential to be cost-saving from a societal perspective and cost-effective from the from the payer perspective, with an ICER of 1,140 OMR (2,964 USD) per QALY saved.

The first estimates of health care and economic burden of severe rotavirus infections in pediatric patients in Oman were provided in a 2009 study by Al Awaidy et al. Their burden estimates indicated that the Omani government spent an estimated 791,817 USD and 1.8 million USD annually to treat rotavirus-associated diarrhea in outpatient and hospital settings, respectively. The current model estimates that RGE-related costs of care are projected to be 2,023,038 OMR (5,259,900 USD) and confirming the substantial burden of severe rotavirus on the health care system in Oman.

Al Awaidy et al. indicated the potential positive impact of rotavirus vaccinations on the substantial burden associated with RGE in Oman. Growing evidence from countries where these vaccinations have been introduced suggests an association with reduced hospitalizations and deaths among children under age [1,28-31]. According to recent reports from Europe, Australia and the United States, reductions of 70% to 95% in the number of hospitalizations for rotavirus-related diarrhea and 35% to 48% for all cause-related diarrhea occurred after the introduction of the vaccine into routine immunization programs [29-31]. These results are consistent with the 89% reduction in the number hospitalizations following implementation of a universal vaccination program in Oman, as predicted in the current study. Oman would be considered a high-income country based on its per capita GDP, so parallels to other high-income countries are relevant.

In the current study, although no multiple scenarios were explored to determine the cost effectiveness of vaccinations at different prices, one-way sensitivity analysis demonstrated that with a vaccine administration fee of 1.44 OMR (3.74 USD), the cost per QALY saved would be 2,051 OMR (5,333 USD) and 534 OMR (1,388 USD) from the payer and societal perspectives, respectively, and thereby cost-effective from both perspectives.

The number of ED visits was not available from the current study, and projections were made based on survey data from Abu Dhabi, UAE, which is a limitation of this study [26]. Furthermore, there were no published data available related to indirect costs of the rotavirus disease; therefore the model assumptions were based on expert opinion.

Despite these limitations, the current analysis strongly supports the introduction of routine rotavirus vaccination in Oman, and provides Omani decision makers with cost effectiveness information on the implementation of a universal vaccination program as part of routine infant immunization programs.

## Conclusions

This cost effectiveness model suggests that a universal RV5 vaccination would have a substantial impact on the reduction of RGE disease burden in Oman and be a cost-effective health care intervention from the payer and cost saving from the societal perspectives. The universal vaccination program would substantially reduce the number of hospitalizations, ED and outpatient visits.

When evaluated from a societal perspective, parental work loss associated with children's RGE episodes is projected to be substantially reduced as a result of universal vaccination. These data serve to inform Omani policy makers in the consideration of a routine rotavirus vaccination within the standard infant immunization program as a cost-effective health intervention.

## Competing interests

BG is a postdoctoral research fellow funded by Merck & Co., Inc. WH and MO are employed by Merck & Co., Inc and own stock in Merck Sharp & Dohme Corp., a subsidiary of Merck & Co., Inc., Whitehouse Station, NJ, the manufacturer of RotaTeq, which is the vaccine referenced in this manuscript. The remaining authors declare that they have no competing interests.

## Authors’ contributions

*Study concept and design:* STA, BG, IA, SA, WH, MO. *Acquisition of data:* STA, BG, IA, SA, WH, MO *Analysis and interpretation of data:* STA, BG, MO. *Drafting of the manuscript*: STA, BG, MO. *Statistical Analysis*: STA, BG, MO. *Critical revision of the manuscript for important intellectual content*: STA, BG, IA, SA, WH, MO. All authors read and approved the final manuscript.

## Pre-publication history

The pre-publication history for this paper can be accessed here:

http://www.biomedcentral.com/1471-2334/14/334/prepub
